# Diagnostic Consistency and Morphological Limits of Extraovarian Lesions in Ovarian Serous Tumors: A Comparative Study Between Gynecological and General Pathologists

**DOI:** 10.3390/diagnostics16081136

**Published:** 2026-04-10

**Authors:** Alina Badlaeva, Anna Tregubova, Natalia Arzhanukhina, Alevtina Chamorovskaya, Vladimir Borzunov, Polina Sheshko, Valentina Litvinova, Larisa Ezhova, Aleksandra Asaturova

**Affiliations:** 1 1st Pathology Department, National Medical Research Center for Obstetrics, Gynecology and Perinatology Named After Academician V.I. Kulakov of the Ministry of Health of Russia, 117513 Moscow, Russia; 2 Department of Pathological Anatomy and Clinical Pathological Anatomy, Pirogov Russian National Research Medical University, 117997 Moscow, Russia; 3 Pathology Department, National Research Center of Oncology Named After N.N. Blokhin, 115522 Moscow, Russia; 4 Pathology Department, Regional Clinical Oncology Hospital, 150054 Yaroslavl, Russia; 5 Pathology Department, Regional Clinical Oncology Dispensary, 460021 Orenburg, Russia; 6 Department of Innovative Oncology and Gynecology, National Medical Research Center for Obstetrics, Gynecology and Perinatology Named After Academician V.I. Kulakov of the Ministry of Health of Russia, 117513 Moscow, Russia

**Keywords:** non-invasive implant, metastasis, extraovarian disease, serous borderline tumor, serous carcinoma low grade, interobserver reproducibility

## Abstract

**Background/Objectives**: Since non-invasive implants and invasive implants (metastases) are a key point of differentiation between serous borderline tumors (SBTs) and low-grade serous carcinoma (LGSC), the correct diagnosis of these two types of extraovarian lesions is crucial for patient treatment and prognosis. However, accurate diagnosis can be challenging even for experienced pathologists. The aim of this study was to evaluate interobserver agreement in the classification of these extraovarian lesions. **Methods**: Twenty-four cases of ovarian SBT and LGSC with 33 samples of non-invasive implants of SBT and metastasis of LGSC were independently reviewed by three gynecologic pathologists and three general pathologists. Diagnostic criteria included destructive invasion, micropapillary architecture, and retraction clefts. To measure interobserver agreement, Fleiss’ kappa and Cohen’s kappa were calculated, with consensus diagnoses determined by the majority of gynecologic pathologists. **Results**: According to the consensus, diagnosis 42.4% biopsies were classified as metastases of LGSC and 57.6% as non-invasive implants of SBT. Overall reproducibility was substantial (κ = 0.61). The agreement among gynecologic pathologists, as well as between gynecologic pathologists and the consensus (using leave-one-out reference), was substantial to near-perfect (κ = 0.745–0.821). General pathologists’ agreement with the consensus was moderate (κ = 0.467–0.698). Agreement between general pathologists was also moderate, with κ values ranging from 0.413 to 0.518. The difference in pairwise agreement between the two groups was statistically significant, confirming that gynecologic pathologists outperformed general pathologists in classifying extraovarian lesions. **Conclusions**: The results showed that current diagnostic reproducibility remains suboptimal, particularly among general pathologists, underscoring the need for improved training and standardized criteria. Ultimately, a multidisciplinary approach combining morphological expertise, immunohistochemical validation and molecular stratification will be essential for optimizing diagnosis and treatment.

## 1. Introduction

Ovarian serous borderline tumor (SBT) is an indolent neoplasm of low malignant potential that typically lacks destructive stromal invasion [1]. The majority of SBTs demonstrate peritoneal implants, which have earlier been classified as either invasive or non-invasive. In the latest WHO (fifth edition) classification of ovarian SBT, the term implant is restricted to extraovarian lesions associated with an SBT. The term invasive implant has now been redefined as metastases of low-grade serous carcinoma (LGSC). Therefore, if a suspected ovarian SBT is found to contain invasive implants, the specimen should be resampled and classified as LGSC [1].

The diagnosis of non-invasive implant versus metastasis has significant prognostic and therapeutic implications. Non-invasive implants are associated with an excellent prognosis, while invasive LGSC metastasis demonstrate increased progression and more aggressive behavior. There are no definitive diagnostic criteria to enable accurate diagnosis of these lesions, which is a frequent reason for differing opinions among pathologists regarding whether metastases of LGSC and non-invasive implants of SBT are distinct entities [2,3]. Accurate classification significantly affects surgical planning, staging, and fertility preservation, especially for young patients, and the need to evaluate and improve diagnostic consistency is critical [4,5].

Thus, knowledge about the interobserver reproducibility for non-invasive implants and metastases among pathologists is limited, and the available data are insufficient and scant. The aim of the current study was to determine the interobserver reproducibility of extraovarian lesions in SBTs and LGSCs among pathologists.

## 2. Materials and Methods

### 2.1. Study Design and Cohort Characteristics

This is a retrospective study of cases with either ovarian SBT or LGSC, as originally diagnosed in the pathology report from departmental in-house and consultation archives of the Research Center for Obstetrics, Gynecology, and Perinatology (Moscow, Russia). To confirm the initial diagnosis, cases were previously reviewed by board-certified gynecological pathologists with more than five years of experience. The inclusion criteria were as follows: histologically confirmed diagnosis of ovarian LGSC and SBT, patients over 18 years of age at the initial diagnosis, and the availability of representative tumor hematoxylin and eosin (H&E) slides. The exclusion criteria were as follows: inadequate or inappropriate tumor samples. Demographic, intraoperative, and clinical follow-up data were obtained from hospital and clinic charts.

A total of 31 consecutive patients with an original diagnosis of ovarian SBT or LGSC and available lesions were identified from the archives of the department. However, 7 cases were excluded because there were no representative H&E slides for the extraovarian lesions. So, this study included 33 specimens of extraovarian lesions from 24 eligible female patients who had undergone surgical treatment in the calendar years 2017–2024.

The dataset contained H&E-stained whole-slide images (WSIs) with a total of 29.5 GB storage. Slides were scanned using a standard WSI scanner Aperio AT2 (Leica Microsystems, Heerbrugg, Switzerland) at 40× magnification (resolution 0.25 µm/pixel), and were subsequently anonymized and randomly assigned.

### 2.2. Histopathological Assessment of Implants

A series of WSIs were rereviewed independently by 3 experienced gynecological pathologists and 3 general pathologists and classified as metastasis or non-invasive implants using criteria according to McKenney et al. (2016) [2]. The morphologic features were as follows: destructive invasion, micropapillary architecture, and clefts.

WSIs were examined using a digital pathology image viewing platform, which is used in the contributing authors’ institution. All the participating pathologists were trained in the ability to distinguish between metastasis or non-invasive implants using example images obtained from the publication conducted by McKenney et al. at the Stanford University School of Medicine [2].

No initial diagnosis, diagnoses assigned by other reviewers, or clinical or demographic information was available to the reviewers during the assessment. Because of the subspecialty expertise of gynecologic pathologists, they might have been able to identify some of the institutional cases. To limit this potential bias, the authors removed all identifying information from the slide labels and file names and randomized the case order.

### 2.3. Statistical Methods

Statistical analyses were performed using GraphPad Prism 9.3.1 (GraphPad Software, San Diego, CA, USA). For indicators, the median (Me) and 25% and 75% quartiles (Q1, Q3) were calculated. Nominal data were summarized descriptively as absolute numbers and percentages. To compare continuous variables such as age and follow-up between the two groups (SBT and LGSC), the Mann–Whitney U test was used with a Bonferroni correction. To compare categorical data such as FIGO staging, Fisher’s exact test was performed.

To measure interobserver agreement, Fleiss’ kappa and Cohen’s kappa were calculated, where a k value of 0 to 0.4 indicates no or minimal agreement, 0.4 to 0.6 moderate agreement, 0.6 to 0.8 substantial agreement, and 0.8 to 1 near-perfect agreement [6]. For the validation of reproducibility, the consensus diagnosis was determined by the majority of experienced gynecological pathologists (2 of 3). To avoid circularity in assessing the level of agreement between each gynecologic pathologist and the consensus, the leave-one-out approach was used. For Fleiss’ kappa, the 95% confidence interval (CI) was calculated using the nonparametric bootstrap method.

Patient survival analysis was performed using the Kaplan–Meier method, and the results were modeled via the log-rank test.

A *p*-value < 0.05 was considered significant.

## 3. Results

### 3.1. Interobserver Reproducibility of Extraovarian Lesions

On the basis of the abovementioned morphological features, 42.4% (14/33) of biopsies were classified according to the consensus diagnosis as metastases of LGSCs and 57.6% (19/33) as non-invasive implants of SBTs.

Interobserver reproducibility for distinguishing non-invasive implants and metastasis was carried out with independent review results by six pathologists with different experience in the gynecological field. The Fleiss’ kappa value for the assessment of invasion was 0.61 (95% CI 0.50–0.71), indicating substantial interobserver agreement. Reviewers’ assessments of the diagnoses are presented in Appendix A.

Nevertheless, according to Table 1, the results of Cohen’s Kappa between pathologists varied from 0.348 to 0.817, and between reviewer and consensus diagnosis from 0.467 to 0.821.

What stands out in Table 1 is the variability of reproducibility among gynecological and general pathologists. The agreement among gynecologic pathologists, as well as between gynecologic pathologists and the consensus (using leave-one-out reference), was substantial to near-perfect, with Cohen’s kappa values ranging from 0.745 to 0.821. General pathologists’ agreement with the consensus was moderate, with κ values ranging from 0.467 to 0.698. Agreement between general pathologists was also moderate, with κ values ranging from 0.413 to 0.518.

The difference in pairwise agreement between the two groups was statistically significant (*p* = 0.008), confirming that gynecologic pathologists outperformed general pathologists in classifying extraovarian lesions.

The interobserver agreement for distinguishing specific histopathological patterns of invasion is summarized in Table 2. Diagnostic concordance varied depending on the morphological pattern assessed.

**Figure 1:**
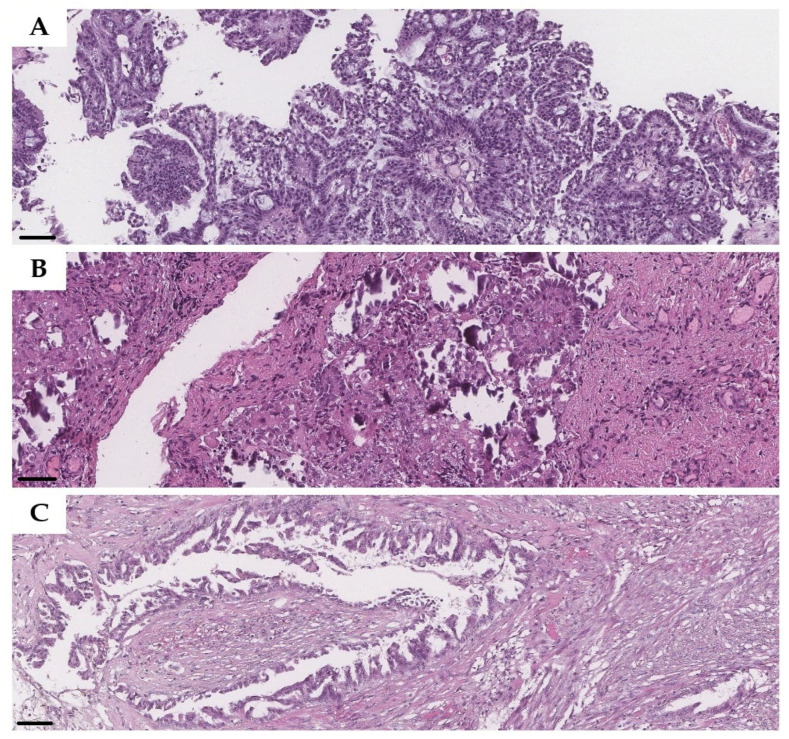
Ambiguous cases of metastasis. (**A**) Micropapillary architecture mistaken for non-invasive implants; (**B**) clefts and micropapillary structures with multiple psammoma bodies (mixed pattern) mistaken for non-invasive implants; (**C**) destructive pattern considered as a non-invasive desmoplastic implant. H&E staining, magnification 20×, resolution 0.25 µm/pixel. Scale bars = 100 µm.

Destructive invasion and retraction artifact (clefts) patterns generally showed perfect agreement among all reviewers. Nevertheless, interobserver agreement was lower for micropapillary architecture, with one such case achieving only 66.7% consensus (Figure 1A). Cases exhibiting a mixed pattern of invasion demonstrated variable results, with rates ranging from 83.3% to 100% depending on the specific combination of features (Figure 1B). Notably, one biopsy classified as showing a destructive pattern also yielded an agreement rate of only 66.7% (Figure 1C), indicating that even this typically well-agreed-upon feature can be a source of diagnostic discrepancy in select, potentially ambiguous, cases.

These results indicate that while pathologists showed high consensus in recognizing classic destructive invasion and retraction artifacts overall, agreement was lower for micropapillary features and in certain individual cases, highlighting specific patterns and challenging morphologies as potential sources of diagnostic variability.

### 3.2. Clinicopathological Characteristics of the Cohort

The clinicopathological characteristics of the study groups are presented in Table 3. After rereviewing biopsies according to the consensus diagnosis, in 13 of 24 patients, the tumor was classified as an SBT and in 11 of 24 patients, the tumor was classified as LGSC.

As can be seen from Table 3, patients in the SBT group were younger than patients in the carcinoma group (Me = 31, Me = 33, respectively), but these differences were not statistically significant. The International Federation of Gynecology and Obstetrics (FIGO) stage was known for all patients. Staging showed that most cases in both groups were advanced (stage III), with 75% of SBTs and 82% of LGSCs. One case of LGSC showed stage IV with multiple liver metastasis. Early stage disease (stage II) with fallopian tube involvement was rare, accounting for 15% (2/13) and 9% (1/11) for each group, respectively.

### 3.3. Histopathological Assessment of Implants

**Figure 2:**
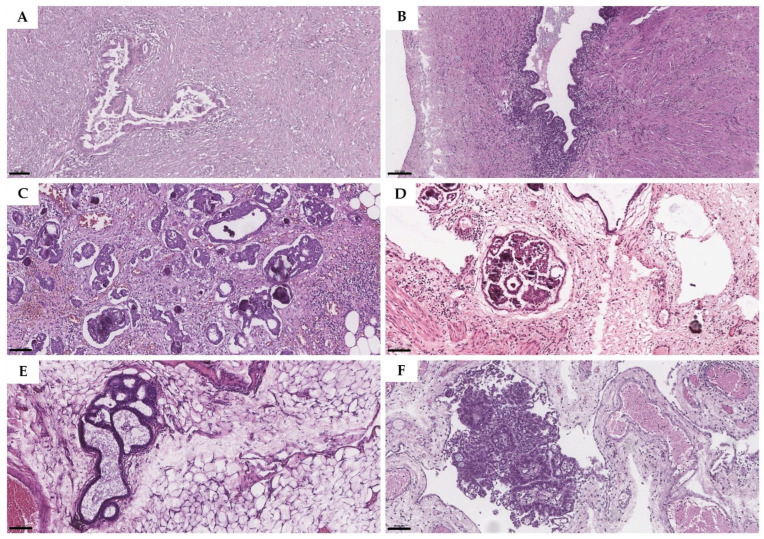
Histological lesions of omentum and peritoneum biopsies in SBT and LGSC groups. (**A**) Destructive pattern of invasion with glands and clusters of tumor cells percolating through the stroma; (**B**) endometriosis with ectopic located endometrial glands and endometrial type stroma; (**C**) metastasis with small isolated papillas within clear lacunar spaces (retraction artifacts); (**D**) non-invasive implant of SBT with micropapillary structures within epithelium lined space; (**E**) endosalpingiosis; (**F**) metastasis with micropapillary architecture. H&E staining, magnification 10×, resolution 0.25  µm/pixel. Scale bars = 100 µm.

The overall series of 14 metastases of LGSC showed the following characteristics (Table 1). Within the aforementioned cohort, histoarchitectural assessment revealed several invasive growth patterns that were found alone or in combination. One of the most commonly detected single patterns of omentum and peritoneum metastases was a destructive variant with glands and clusters of tumor cells percolating through the stroma (Figure 2A). In two biopsies, differential diagnosis included foci of endometriosis (Figure 2B).

Another frequent type of metastasis was clefts in fibrotic stroma with nests of epithelial cells surrounded with retraction artifacts (Figure 2C). In contrast, non-invasive implants showed multiple micropapillary structures confined within an epithelium-lined space (Figure 2D). Also, in cases of SBT, endosalpingiosis was found (Figure 2E). In total, 7.1% (1/14) metastases demonstrated pure micropapillary architecture (Figure 2F). The mixed architecture was identified in five cases, with three samples demonstrating a combination of micropapillary and destructive patterns; the other two showed both micropapillary structures and retraction artifacts.

### 3.4. Correlation of Tumor Type with Clinical Follow-Up

Prognosis analysis was performed on the disease-free survival (DFS) time of 23 patients (for 13 SBT and for 10 LGSC), with a median follow-up period of 45 months. The log-rank test and Kaplan–Meier survival curve were employed to evaluate the correlation between the type of extraovarian lesion and DFS of patients.

**Figure 3:**
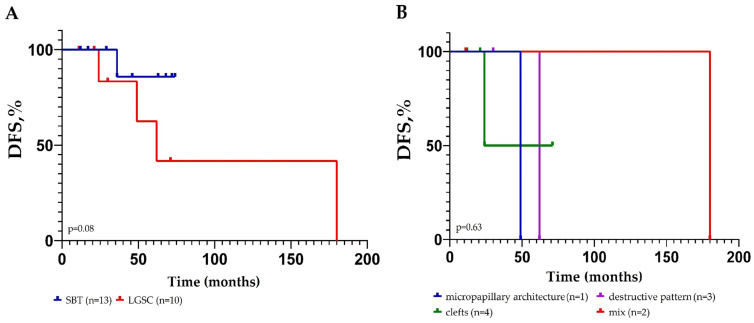
Univariable analysis of disease-free survival (DFS). (**A**) DFS of patients with SBT (*n* = 13) and LGSC (*n* = 10). (**B**) DFS of 10 patients with different morphological patterns of metastasis.

As can be seen from the Kaplan–Meier curves (Figure 3A), analysis of disease-free survival did not identify any statistical differences between extraovarian lesions of SBTs and LGSC (*p* = 0.08).

Of the 13 patients with non-invasive implants, 11 of them were alive, with no evidence of disease and follow-up ranging from 12 to 72 months, and only one had tumor recurrence in 36 months. Of the 10 patients with metastasis, two were alive with progressive disease in 49 and 62 months. In one of the patients, local tumor recurrence was identified 181 months after the initial diagnosis (primary tumor in 2008, recurrence in 2023). One patient died in 2 years, and six were alive with no evidence of disease with follow-up ranging from 11 to 71 months.

DFS between metastasis subtypes (invasion patterns) was not compared, as the number of patients with each pattern was extremely small (e.g., pure micropapillary pattern—one case). The *p* values in the Kaplan–Meier curves (Figure 3B) for each group are provided for illustrative purposes only due to insufficient power.

Of the 10 patients with metastasis depending on different types of invasion, the following results were found. In the single case of a pure micropapillary pattern, the patient was alive with progressive disease in 49 months. In one case with clefts, the patient died in 2 years, and three were alive with no evidence of disease after 12, 21, and 71 months. In one of the patients with destructive invasion, local tumor recurrence was identified after 62 months; two were alive without progressive disease after 12 and 30 months. In one of the patients with a mixed pattern of invasion, local tumor recurrence was identified 181 months after the initial diagnosis (primary tumor in 2008, recurrence in 2023) and one was alive with no evidence of disease in 12 months.

## 4. Discussion

Since non-invasive implants and invasive implants (metastasis) are a key point of differentiation between SBT and LGSC, the correct diagnosis of these two types of extraovarian lesions is crucial for patient treatment and prognosis. According to the NCCN, there are some key differences between SBT and LGSC in the surgical context, staging requirements, and lymph node dissection [7]. Therefore, only an accurate diagnosis of metastasis can prevent excessive surgery and spare fertility in young patients.

In the current study, the results of the agreement among all reviewers showed that Fleiss’ kappa was 0.61, indicating substantial reproducibility in the estimation of extraovarian lesions between observers. Nevertheless, gynecologic pathologists showed substantial to near-perfect Cohen’s kappa values, while the results for general pathologists were moderate.

An important observation from this study is the considerable difference in performance between gynecologic and general pathologists. Gynecologic pathologists demonstrated near-perfect agreement with the consensus panel (κ = 0.745–0.821), while general pathologists showed only moderate to substantial agreement (κ = 0.467–0.698), with no overlap in ranges. This difference is likely due to the relative frequency with which gynecologic and general pathologists encounter ovarian serous tumors and their extra-ovarian manifestations. General pathologists may be less familiar with subtle histological features, especially micropapillary architecture and clefts.

Our data showed lower overall reproducibility than previous studies. For example, McKenney et al. reported a kappa value of 0.71–0.84, depending on the characteristics of the metastasis studied (181 patients included) [2], and Mhawech-Fauceglia et al. reported excellent inter-rater agreement, with a kappa around 0.85 (although the exact data were not provided and we calculated it for comparison); however, the cohort was small (only 23 cases) [8]. These differences can be predominantly explained by the inclusion of more participating researchers in the current study, with varying levels of pathology expertise in this field. Other authors also note that although histopathologic examination is reliable and reproducible for experienced gynecologic pathologists, it can occasionally be difficult, especially when the biopsied lesion is small [9].

The lower interobserver reproducibility may also be due to the difficulty of distinguishing extraovarian lesions based on morphologic criteria, which can be challenging even with established guidelines. This difficulty may lead to a lower reproducibility rate, especially among individuals less familiar with this specific gynecologic pathology. The higher reproducibility reported in other studies may be related to the reviewers’ greater level of training regarding ovarian tumors. Discordant results in interobserver reproducibility could also be attributed to a heterogeneous cohort and small sample size. Differences in the “difficulty” of the cases used (for example, more ambiguous morphologies) compared to other studies could also affect the level of agreement. A cohort consisting of more classic, unambiguous cases would yield higher kappa values.

Outside the spectrum of SBT-LGSC, two benign entities have been found to mimic extraovarian lesions. Endometriosis was correctly identified by all reviewers. However, the glandular component may raise concern for carcinoma, especially if no other tissue is available [10,11,12]. Endosalpingiosis, characterized by several empty spaces lined by ciliated tubal-type epithelium, was also present. This condition may be confused with non-invasive implants, especially if papillary morphology is not evident [13,14,15]. Another mimic, particularly of metastatic carcinoma and non-invasive implants, is the rare papillary type of endosalpingiosis [16].

In our study, the analysis of DFS revealed no statistically significant differences between the SBT and LGSC groups (*p* = 0.08). Although the Kaplan–Meier curves demonstrate a discrepancy in favor of SBTs, the interpretation of this result requires caution. First, the sample size was insufficient to achieve statistical power (only 23 patients, of whom only four relapsed). Second, the median follow-up was 45 months, which may be insufficient for a full assessment of long-term outcomes, given the indolent nature of LGSC and its tendency to late relapse. Thus, the absence of significant differences should not be interpreted as evidence of a similar prognosis for SBTs and LGSC; on the contrary, the obtained data highlight the need for multicenter studies with standardized diagnostic criteria and long-term follow-up.

It is reported that destructive growth was directly associated with poor prognosis, but metastases with micropapillary architecture and clefts had almost the same prognosis as SBTs [2]. The study conducted by Varghese and Shih also noted the different clinical courses of metastases with various histomorphological patterns [9]. In a recent study of Chinese breast cancer patients, retraction clefts were found to have limited prognostic value and were present in only 15.5% of cases, much lower than reported in Western studies [17]. This suggests that the significance of retraction clefts may not be universal in assessing cancer invasion and aggressiveness. In cases of LGSC, where the presence of retraction clefts is one of the diagnostic criteria, such reliance may not be justified.

Consequently, the reproducibility of serous tumor extraovarian lesions still needs improvement to reach excellence, especially among general pathologists. Unfortunately, additional methods have little impact on differential diagnosis. For example, Lee et al. showed that the combined sensitivity and specificity of calretinin, CD34, and α-SMA were 100% and 81%, respectively. However, they noted that this method may not be helpful for small biopsies of non-invasive desmoplastic implants [18]. So, they concluded that an exact differentiation of peritoneal “implants” as metastases of ovarian carcinomas or autochthonous neoplasms in the context of multifocal tumorigenesis is not possible on the basis of their immunohistochemical findings.

These data suggest that further investigation is necessary to improve the accuracy and clinical relevance of diagnostic features for invasiveness, as a solitary invasive implant of an SBT does not exhibit the same clinical behavior as widespread LGSC, even though they are formally classified within the same advanced LGSC group (stage III/IV) [19]. It has also been shown that extraovarian lesions of serous tumors differ not only histologically but also molecularly. RAS-related mutations appear to be among the most important steps in the transition from SBT to LGSC [20]. Initially, it was assumed that *BRAF* and *KRAS* mutations are mutually exclusive and do not occur in implants [21,22]. It was later demonstrated that the *KRAS* mutation, but not the *BRAFV600E* mutation, is significantly associated with metastasis and poor prognosis [23,24]. However, *KRAS* mutations can be detected in tumors with both metastasis and non-invasive implants, so this test cannot be used for the precise differential diagnosis of SBTs and LGSC [25].

Therefore, more extensive molecular testing is required to investigate the nature of extraovarian lesions. Mhawech-Fauceglia et al. proposed an eight-gene panel as a potential tool to refine prognosis and therapy [8]. According to this clustering, non-invasive implants exhibit anti-tumor signaling pathways, while metastases mimic high-grade serous carcinomas (HGSC) with DNA repair dysregulation and cell cycle hyperactivity. Furthermore, these results confirm that metastases are molecularly heterogeneous, with a subset harboring HGSC-like signatures.

Several limitations of this pilot study should be acknowledged. First, the results are based on a small sample size, partly due to the rarity of LGSCs [1,2,3]. Similarly, most previous studies have also been limited to a small number of cases [2,3,8,18]. Additionally, the use of a restricted retrospective cohort from departmental in-house and consultation archives may introduce sampling bias and limit the statistical power and generalizability of our findings. Although the WSIs were anonymized, the two groups of pathologists differed not only in subspecialty training but also in familiarity with the digital platform; this may have differentially affected their performance. To mitigate this, all reviewers underwent a brief calibration session using example images before the assessment. Moreover, the data should be interpreted with caution because this investigation relies entirely on histomorphological criteria for classification, without integrating ancillary techniques such as immunohistochemistry or molecular profiling into the diagnostic algorithm, which might have improved accuracy or provided explanatory insights for disagreements. Additionally, the current research was unable to analyze crosstalk between type of extraovarian lesion and clinical follow-up, as well as variable duration, limiting the ability to perform a robust, long-term survival analysis correlated with specific morphological patterns of invasion.

Nevertheless, our findings highlight once again the diagnostic reliability essential for effective clinical management. The distinction between metastases and non-invasive implants must become more central in determining treatment pathways. Younger patients with non-invasive disease can confidently be offered fertility-sparing surgery with unilateral salpingo-oophorectomy and full peritoneal staging, and this approach can achieve both oncological and quality of life goals [26,27]. However, the presence of metastatic LGSC changes the surgical staging requirements to those for invasive carcinoma, including hysterectomy, bilateral salpingo-oophorectomy, omentectomy, and lymphadenectomy [28,29,30]. The diagnostic variability described here, particularly among general pathologists, carries the risk of underdiagnosing LGSC or overdiagnosing an SBT. Both scenarios have significant implications for survival and fertility, with differing degrees of impact. A pathologist should conduct a secondary review of all SBT cases with extraovarian disease, a standard practice that should be incorporated into clinical guidelines.

While our study highlights a concerning disparity in diagnostic reproducibility, particularly between general and specialized pathologists, it also identifies several key directions for the future. Firstly, developing and disseminating standardized diagnostic algorithms and digital training modules could significantly improve reproducibility. The adoption of WSIs and AI-assisted pattern recognition may further reduce subjectivity in evaluating ambiguous features, such as micropapillary architecture or stromal invasion. Secondly, molecular profiling, including mutational analysis of *KRAS*, *BRAF*, and gene expression signatures, should be explored as an adjunct, not a replacement, to histology in selected diagnostically challenging cases. A multidisciplinary approach combining morphological expertise, immunohistochemical validation and molecular stratification will be essential for optimizing the diagnosis and treatment of peritoneal implants to ensure tailored therapies that balance oncological safety and quality of life, especially for young patients who wish to preserve their fertility. Moreover, reliance on less predictive morphologic patterns should be minimized, and standardized training should be implemented with improved training programs for pathologists, particularly to distinguish subtle histological features.

## 5. Conclusions

The precise distinction between metastasis and non-invasive peritoneal implants in ovarian LGSC and SBT is crucial for patients, as they differ greatly in terms of surgical approach, staging requirements and prognosis. While non-invasive implants allow for conservative, fertility-sparing treatments with excellent outcomes, metastasis requires aggressive surgical and staging protocols, as with invasive cancers. However, current diagnostic reproducibility remains suboptimal, particularly among general pathologists, underscoring the need for improved training and standardized criteria. In the future, diagnostic criteria should be refined with a focus on destructive invasion as the primary prognostic feature.

## Figures and Tables

**Table 1:** Pairwise agreement values.

	Path 1	Path 2	Path 3	Path 4	Path 5	Path 6
**Path 1**		0.812	0.817	0.639	0.619	0.530
**Path 2**			0.756	0.698	0.687	0.348
**Path 3**				0.697	0.573	0.578
**Path 4**					0.518	0.513
**Path 5**						0.413
**CD**	0.821	0.745	0.781	0.698	0.687	0.467

Pathologist (Path); Consensus diagnosis (CD); Path 1–3 are gynecological pathologists and Path 4–6 are general pathologists.

**Table 2:** Interobserver agreement for distinguishing specific histopathological patterns of invasion.

Micropapillary	Clefts	Destructive Pattern	% of Agreement
1	0	0	66.7
1	0	1	100
0	1	0	100
0	0	1	66.7
0	1	0	83.3
0	0	1	83.3
0	1	0	100
1	0	1	83.3
1	0	1	100
0	0	1	100
1	1	0	100
1	1	0	100
0	1	0	83.3
0	0	1	100

0—absence of feature; 1—presence of feature.

**Table 3:** Clinicopathological features of SBT and LGSC.

Characteristics	SBT (*n* = 13)	LGSC (*n* = 11)	*p*-Value
Median (Q1; Q3)			
Age (yr)	31 (27; 38)	33 (29; 33)	0.57
Follow-up (mn)	36 (23; 65.5)	27 (12; 64.2)	0.16
FIGO stage:		*n* (%)	
II B	2 (15)	1 (9)	0.58
III A	8 (62)	5 (46)	
III B	3 (23)	3 (27)	
III C	0	1 (9)	
IV	0	1 (9)	
Pattern of invasion:	*n* (%)	*	
micropapillary architecture	N/A	1 (7)	N/A
retraction artifact	N/A	4 (28.5)	
destructive pattern	N/A	4 (28.5)	
mixed	N/A	5 (36)	

Serous borderline tumor (SBT); low-grade serous carcinoma (LGSC); International Federation of Gynecology and Obstetrics (FIGO); not applicable (N/A). * Values are given for 14 of 33 biopsies, which were classified according to the consensus diagnosis as metastases of LGSC.

## Data Availability

The data are not publicly available due to privacy reasons. The data that support the findings of this study are available from the corresponding author [A.B.] upon reasonable request.

## Abbreviations

The following abbreviations are used in this manuscript:

SBT	Serous Borderline Tumor
LGSC	Low-Grade Serous Carcinoma
H&E	Hematoxylin and Eosin
WSI	Whole-Slide Image
Me	Median
Q1	First Quartile (25th percentile)
Q2	Third Quartile (75th percentile)
FIGO	International Federation of Gynecology and Obstetrics
N/A	Not Applicable
DFS	Disease-Free Survival
CI	Confidence Interval
Path	Pathologist
CD	Consensus Diagnosis
NCCN	National Comprehensive Cancer Network
HGSC	High-Grade Serous Carcinoma
AI	Artificial Intelligence

## Appendix A

**N Case**	**Path 1**	**Path 2**	**Path 3**	**Path 4**	**Path 5**	**Path 6**	**CD**
1	1	1	1	1	1	2	1
2	1	1	1	1	1	1	1
3	1	1	1	1	1	1	1
4	1	1	1	1	1	1	1
5	1	1	1	1	1	1	1
6	2	2	2	2	1	1	2
7	2	2	2	2	2	2	2
8	1	1	1	1	1	1	1
9	2	2	2	2	2	2	2
10	1	2	2	2	2	1	2
11	1	1	1	2	1	2	1
12	1	1	1	1	1	1	1
13	1	1	1	1	1	1	1
14	1	1	2	2	1	2	1
15	1	1	1	1	2	2	1
16	2	2	2	2	1	2	2
17	1	2	1	2	2	1	1
18	1	1	1	1	1	2	1
19	1	1	2	1	1	2	1
20	2	2	2	1	2	2	2
21	2	2	2	2	2	2	2
22	1	1	1	1	1	1	1
23	1	1	1	2	1	2	1
24	1	1	1	1	1	1	1
25	2	2	2	2	1	2	2
26	1	1	1	1	1	1	1
27	2	2	2	2	2	2	2
28	1	1	1	1	1	1	1
29	2	2	2	2	2	2	2
30	2	2	2	2	2	2	2
31	2	2	2	2	2	2	2
32	2	1	2	2	2	2	2
33	2	2	2	2	2	2	2
Pathologist (Path); consensus diagnosis (CD); Path 1–3 are gynecological pathologists and Path 4–6 are general pathologists; 1—implant of SBT; 2—metastasis of LGSC.							
